# Gait Changes After a Mobile Health Exercise Intervention in Older Adults With Myeloid Neoplasms: Single-Arm Pilot Trial

**DOI:** 10.2196/80909

**Published:** 2026-04-29

**Authors:** Ying Wang, Marielle Jensen-Battaglia, Po-Ju Lin, Ian Kleckner, Elisabeth Hayward, Jason Mendler, Jane Liesveld, Marissa LoCastro, Cait Fallone Sharma, Soroush Mortaz Hedjri, Richard Dunne, Karen Mustian, Kah Poh Loh

**Affiliations:** 1 Department of Public Health Sciences School of Medicine and Dentistry University of Rochester Rochester, NY United States; 2 Department of Surgery University of Rochester Medical Center Rochester, NY United States; 3 Department of Pain & Translational Symptom Science School of Nursing University of Maryland, Baltimore Baltimore, MD United States; 4 School of Medicine and Dentistry University of Rochester Rochester, NY United States; 5 Division of Hematology/Oncology, Department of Medicine James P. Wilmot Cancer Institute University of Rochester Rochester, NY United States; 6 Department of Internal Medicine University of Wisconsin Madison, WI United States; 7 James P. Wilmot Cancer Institute Rochester, NY United States

**Keywords:** exercise intervention, gait, mobile health, older adults, myeloid neoplasms

## Abstract

**Background:**

Myeloid neoplasms (MNs) are most frequently diagnosed among adults aged 60 years and older. Cancer and chemotherapy can cause gait disturbances and increase fall risk in older adults with MNs. Exercise may improve gait, but there is a lack of research among older adults with MNs undergoing active chemotherapy.

**Objective:**

We explored gait changes following a home-based mobile health exercise intervention during 2 cycles of outpatient chemotherapy (8-12 weeks).

**Methods:**

In a single-arm pilot study, we included adults aged 60 years and older with MNs undergoing chemotherapy. Geriatric Oncology-Exercise for Cancer Patients intervention integrates a progressive aerobic walking and resistance exercise program with a mobile app. We assessed gait by using a waist-worn G-Walk motion sensor during a 6-minute walk at the preintervention and postintervention time points. Spatiotemporal outcomes included cadence (steps per minute), velocity (meters per minute), normalized stride (stride length normalized over height), and swing duration (percentage of the gait cycle during which a foot is in the air when walking). Regularity outcomes that measure gait rhythm included variability of normalized stride and variability of swing duration. Variability for both outcomes was quantified as the SD across all gait cycles. We calculated Cohen *d* effect sizes (ESs) for change in gait outcomes and used the Spearman rank correlation to correlate changes in daily steps and resistance exercise duration with gait outcomes.

**Results:**

We included 13 patients (mean age 71, SD 4.8 years); most were male (n=8, 61.5%), White individuals (n=12, 92.3%), and non-Hispanic individuals (n=13, 100%). Average daily steps were 3084 (SD 1765.5) at the preintervention time point and 3757 (SD 2623.6) at the postintervention time point. Patients performed resistance exercises for 25 minutes per day, 4 days per week at low intensity (mean rating of perceived exertion 3/10, SD 1.3). At the postintervention time point, we observed numerical changes in gait outcomes, including increased cadence (mean +4.6, SD 14.6 steps per minute; *P*=.24; ES=0.38) and decreased variability in normalized stride (mean −1.4%, SD 8.5%; *P*=.34; ES=−0.18) and swing duration (mean −0.1%, SD 1.1%; *P*=.54; ES=−0.15), although these improvements were not statistically significant. Increased daily steps significantly correlated with decreased swing duration variability (*r*=−0.72; *P*=.01). Resistance exercise duration significantly correlated with increased cadence (*r*=0.54; *P*=.06) and velocity (*r*=0.56; *P*=.05).

**Conclusions:**

In our exploratory analyses, better adherence to exercise correlated with improved gait outcomes. Our ongoing pilot randomized controlled trial (ClinicalTrials.gov identifier: NCT04981821) will further examine the effects of the Geriatric Oncology-Exercise for Cancer Patients intervention on gait outcomes in this population.

## Introduction

Gait is an important indicator of overall health and functional status [1-3]. Although walking speed is a well-recognized marker of many health outcomes, it arises from more granular aspects of gait, including both spatiotemporal measures (eg, gait speed, stride length, cadence, and symmetry) and regularity measures (eg, variability of stride length) [1,4,5]. Cancer and its treatments can contribute to disturbances in these more detailed aspects of gait long before measurable loss in gait speed or function [6]. Possible pathways include musculoskeletal effects, including fatigue and sarcopenia, which influence body movement, and neurological effects, including direct tumor infiltration or compression and chemotherapy-induced neurotoxicity [6]. For example, chemotherapy-induced peripheral neuropathy in older adults with cancer is commonly associated with shorter step length, loss of symmetry or synchrony, and greater step width variability in addition to slower gait speed [3,7]. Gait disturbances have been linked to poor clinical outcomes, including falls, unplanned hospitalizations, emergency department visits, and mortality [2,3]. While habitual physical activity may mitigate some adverse effects of cancer and its treatments, gait disturbances still occur in older adults with cancer [8]. Therefore, there is a need for interventions that can help maintain normal gait in this population.

Myeloid neoplasms (MNs; eg, acute myeloid leukemia [AML] and myelodysplastic syndrome [MDS]) are most frequently diagnosed among adults aged 60 years and older [9]. Older adults with MNs are vulnerable to gait disturbances due to the combined effects of aging, cancer, and treatment-induced toxicities [3,7,10]. Despite the growing number of studies on exercise interventions in older adults with cancer, the impact of specific exercise programs on gait outcomes in older adults with MNs undergoing active chemotherapy is understudied. We previously established the feasibility and usability of a single-arm mobile health exercise program for older adults with MNs [11]. Using data from this pilot study, we aimed to explore (1) changes in gait outcomes following exercise and (2) correlations of exercise adherence with changes in gait outcomes.

## Methods

### Study Design, Setting, and Participants

We conducted a single-arm pilot study at the University of Rochester Medical Center (ClinicalTrials.gov identifier: NCT04035499) to evaluate the feasibility of a mobile health exercise intervention, Geriatric Oncology-Exercise for Cancer Patients (GO-EXCAP), for older adults with MNs. Patients were screened and recruited between February 2020 and July 2021. Eligible patients were approached after confirming with their treating oncology team. Eligibility criteria were (1) age of 60 years and older; (2) being English speakers; (3) diagnosis of an MN, such as AML, MDS, or MDS and myeloproliferative neoplasm overlap; (4) undergoing outpatient chemotherapy; (5) a physician-verified Eastern Cooperative Oncology Group (ECOG) performance status of 0 to 2; (6) ability to walk 4 m; and (7) capacity to provide informed consent. Individuals were excluded from the study if they (1) had medical contraindications to exercise as determined by their treating oncologists, (2) had a low platelet count on the most recent complete blood count (ie, ≤10,000 platelets per microliter), and (3) did not receive platelet transfusion (eg, due to platelet refractoriness). Findings from the primary study have been previously reported [11].

This study is a secondary analysis of data from the parent study [11]. We report this analysis following the TREND (Transparent Reporting of Evaluations With Nonrandomized Designs) guidelines for nonrandomized evaluations of behavioral and public health interventions (Table S1 in Multimedia Appendix 1).

### Intervention and Study Procedures

GO-EXCAP is a mobile health exercise intervention developed to promote successful behavior change by using evidence-based approaches to improve exercise self-efficacy (ie, verbal persuasion, social modeling, imaginal experiences, and provision of regular monitoring and feedback on mastery as well as emotional and physiological states).

GO-EXCAP integrates the registered and copyrighted home-based exercise intervention Exercise for Cancer Patients (EXCAP) with the GO-EXCAP mobile app. The EXCAP intervention was designed and delivered by American College of Sports Medicine–certified clinical exercise physiologists and has been tested in multiple studies [12-14]. It consists of progressive aerobic walking and resistance band exercise prescribed by a clinical exercise physiologist during a one-on-one in-person teaching session. The exercise physiologist generated individually tailored exercise prescriptions for each participant, increasing their baseline daily steps weekly by 5% to 20%. Between 5% and 20% weekly increase in steps would allow individuals classified as sedentary (ie, <5000 steps) to progress toward or achieve an active classification (ie, ≥10,000 steps) over an 8- to 12-week intervention period. The 5% to 20% step progression rate was individualized based on three key criteria: (1) average daily step counts at baseline, (2) participants’ physical capability and perceived readiness, and (3) weekly performance during the intervention. The strategy used to determine the weekly progression rate was described in Multimedia Appendix 2. For resistance band exercise, participants also received individualized prescriptions based on their baseline fitness level. The protocol began with 1 set of 8 to 15 repetitions for 16 exercises, using color-coded resistance bands appropriate for individual strength. Under the instruction of exercise physiologists, participants progressed weekly by increasing sets and repetitions until reaching 4 sets of 15 repetitions. Upon reaching this milestone, participants transitioned to a heavier resistance band and repeated the progression. Throughout the intervention, participants were instructed to perform both aerobic walking and resistance band exercises at a low to moderate intensity, corresponding to a rating of perceived exertion (RPE) of 3 to 5 on a scale from 1 to 10 (1=“not tired at all”; 10=“extremely tired”) [15]. We provided participants with a GO-EXCAP kit containing a printed manual, an activity tracker to record daily steps (Garmin Forerunner 35), 3 therapeutic bands with resistance levels corresponding to 2 to 14 pounds, and a tablet preloaded with the GO-EXCAP app. The exercise physiologist monitored participant-entered exercise data on the app and adjusted prescriptions as needed.

The EXCAP intervention lasted 6 weeks in prior trials [12-14]. Because chemotherapy for MNs is typically 4 weeks per cycle, the intervention duration was extended to align with 2 chemotherapy cycles. Given that chemotherapy delays are common in clinical practice, the GO-EXCAP intervention lasted 8 to 12 weeks, or 2 chemotherapy cycles.

### Measures

#### Demographic and Clinical Characteristics

Demographic and clinical characteristics collected at baseline included age, sex, race, ethnicity, marital status, educational attainment, ECOG performance status, cancer type, and chemotherapy cycle at the initiation of the intervention. The ECOG performance status is a validated and widely used scale to assess a patient’s ability to perform daily activities, with scores ranging from 0 (fully active) to 5 (deceased). ECOG scores are associated with various health outcomes in patients with cancer, including emergency department visits, hospitalizations, and mortality [16].

#### Exercise Adherence

During the intervention, daily exercise data collected through the GO-EXCAP app included the number of steps from the activity tracker and self-reported minutes of resistance band exercise and RPE on a scale from 1 to 10 for EXCAP exercises [17]. These metrics provide a comprehensive representation of exercise adherence across exercise type, frequency, duration, and intensity.

#### Gait Outcomes

Participants wore a G-Walk motion sensor (BTS Bioengineering) around the waist while performing the 6-minute walk at baseline and the postintervention time point. All assessments were administered and supervised by clinical exercise physiologists. The exercise physiologists instructed participants to walk as fast as they could on level ground for up to 6 minutes. The G-Walk motion sensor is a noninvasive, lightweight, waist-worn device equipped with a triaxial accelerometer, gyroscope, magnetometer, and sensor fusion. It collects gait data and transmits them to the G-Studio software (BTS Bioengineering) through a Bluetooth connection for functional evaluations of walking. Prior to performing any assessments, all clinical exercise physiologists completed study-specific training and followed standardized operating procedures to ensure consistency and adherence to the protocol. All assessments were periodically audited by a second trained clinical exercise physiologist to ensure procedural fidelity and interrater reliability across exercise physiology personnel.

**Figure 1:**
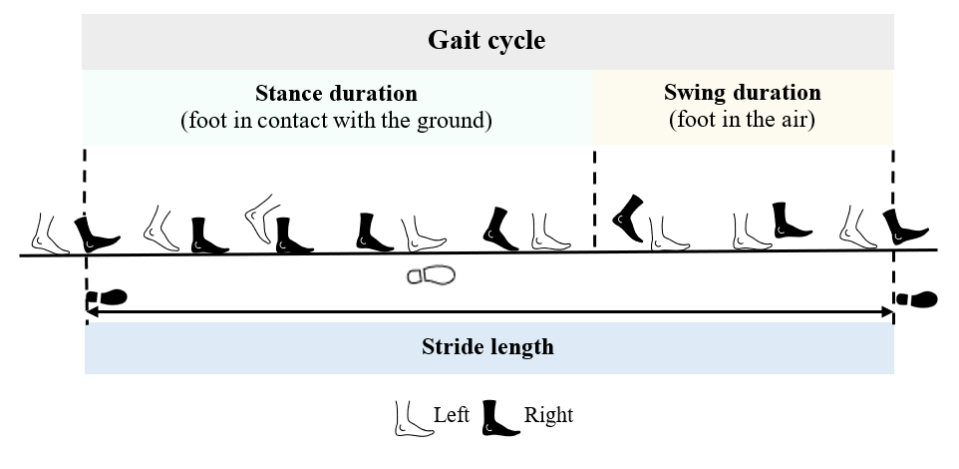
An overview of the walking gait cycle (with the right leg as the reference leg).

Figure 1 describes a gait cycle consisting of stance and swing phases. We measured both the spatiotemporality and regularity of gait (Table 1) [18]. The spatiotemporal outcomes included walking distance covered in 6 minutes (meters), cadence (steps per minute), velocity (meters per minute), stride length (meters), normalized stride (percentage of height), general symmetry index (GSI), stance duration (percentage of gait cycle), and swing duration (percentage of gait cycle). Regularity outcomes that measured gait rhythm included variability of normalized stride (percentage of height) and variability of swing duration (percentage of gait cycle).

**Table 1:** Gait outcomes measured during the 6-minute walk test^a^.

Dimension and outcome		Definition
**Spatiotemporality**		
	Walking distance	The distance covered in 6 min (meters); higher is better
	Cadence	The number of steps per minute; higher is better
	Velocity	The distance covered per minute (meters per min); higher is better
	Stride length	The distance covered from the moment one foot hits the ground to the next time that same foot touches the ground again (meters), equivalent to the length of a full gait cycle; higher is better
	Normalized stride	Normalized over height: calculated as normalized stride length = stride length (meters)/height (meters); higher is better
	General symmetry index	An index ranging from 0% to 100% indicating symmetry between the right and left gait cycles based on the comparison of the antero-posterior acceleration component; higher is better
	Stance duration	The percentage of time for which a foot remains in contact with the ground (optimal range: 60%-62%); bilateral stance duration is calculated using the average of the left and right stance durations
	Swing duration	The percentage of time for which a foot is in the air during walking (optimal range: 38%-40%); bilateral swing duration is calculated using the average of the left and right swing durations
**Regularity**		
	Variability of normalized stride	The SD of stride length across all gait cycles; lower is better
	Variability of swing duration	The SD of swing duration across all gait cycles; lower is better

^a^The variability of stance and swing duration is identical due to the statistical relationship between them (stance + swing duration = 100%). Therefore, we reported only the variability of swing duration in this study.

### Statistical Analysis

Participants with gait outcomes available at both baseline and the postintervention time point were included in the analyses. To evaluate potential selection bias, we compared baseline characteristics between participants included in this analysis and those who were not. For participants included in this analysis, we described their characteristics, exercise adherence, and gait outcomes. Depending on the data distribution, we used paired 2-tailed *t* tests or Wilcoxon signed-rank tests to evaluate within-subject changes in each gait outcome from baseline to the postintervention time point. We also calculated Cohen *d* effect sizes (ESs) to quantify changes from baseline to the postintervention time point (small effect: 0.20 to <0.50; moderate effect: ≥0.50 to <0.80; large effect: ≥0.80) [19]. Spearman correlation analyses were conducted to assess the correlations of changes in daily steps and the duration of resistance band exercise with changes in gait outcomes. Individual data were presented in scatterplots to detect potential influential observations. In sensitivity analysis, we repeated the Spearman correlation analyses after excluding one observation that was more than 1.5 IQRs below the first quartile and did not follow the general trend of the rest of the data.

Given the exploratory nature of this analysis and the small sample size, no imputation methods were applied. A complete-case approach was used for all analyses. We performed hypothesis testing at an α value of .10 (2-tailed). All analyses were conducted using SAS (version 9.4; SAS Institute).

### Ethical Considerations

The parent study was approved by the Research Subjects Review Board of the University of Rochester (study ID: STUDY00003945), and all participants provided informed consent. To ensure participant privacy and confidentiality, all data were deidentified and managed via REDCap (Research Electronic Data Capture; Vanderbilt University), a secure web application accessible only to authorized research personnel. Participants received US $50 in compensation after completing all baseline and postintervention activities. The original ethics approval covers secondary analysis, and thus, no additional consent was required for this study.

## Results

### Participant Characteristics and Exercise Adherence

**Figure 2:**
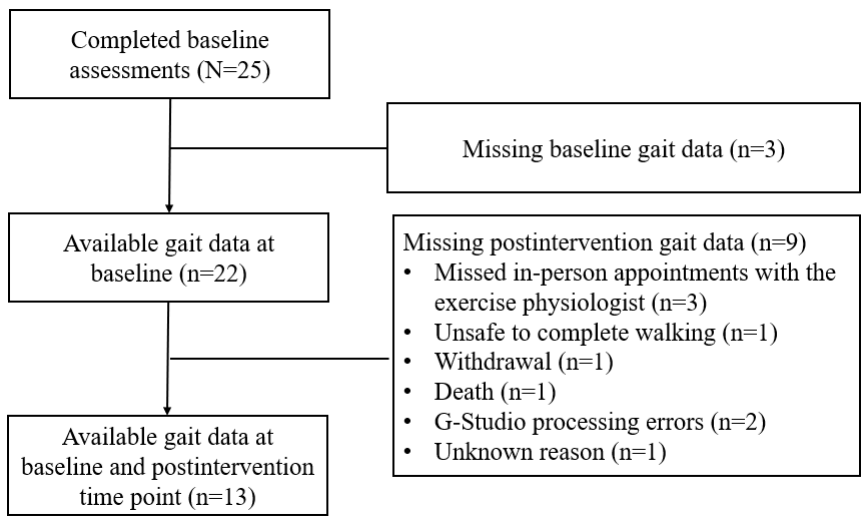
CONSORT (Consolidated Standards of Reporting Trials) flow diagram.

We included 13 participants in this analysis (Figure 2). Of the 25 participants enrolled, 3 (12%) had missing baseline gait data due to logistical issues (eg, Bluetooth connection error). Of the 22 participants with available baseline gait data, 13 (59.1%) completed postintervention gait measures. Reasons for missing postintervention gait data included missed in-person appointments with the exercise physiologist due to health decline (3/22, 13.6%), being unsafe to complete walking (1/22, 4.5%), withdrawal (1/22, 4.5%), death (1/22, 4.5%), G-Studio processing errors (2/22, 9.1%), and unknown reasons (1/22, 4.5%). Comparison of baseline characteristics between participants included in the analysis and those who were not did not reveal substantial differences (Table S1 in Multimedia Appendix 3).

Table 2 describes the characteristics and exercise adherence of the 13 participants with both baseline and postintervention gait data. These participants had a mean age of 71 (SD 4.8) years, and most were White (n=12, 92.3%) and non-Hispanic (n=13, 100%) individuals. More than half were male (n=8, 61.5%), married (n=9, 69.2%), and college graduates or of higher educational levels (n=7, 53.8%). Most participants were diagnosed with AML (n=7, 53.8%) or MDS (n=5, 38.5%). These 13 participants had a mean of 3084 (SD 1765.5) daily steps at baseline and 3757 (SD 2623.6) daily steps at the postintervention time point. On average, they performed resistance band exercises for 4 days per week, 25 minutes per day, with a mean RPE of 3/10 (SD 1.3). No adverse events were reported related to the intervention [11]. Overall, this intervention demonstrated excellent tolerability among older adults with MNs.

**Table 2:** Demographic and clinical characteristics (n=13).

Variable		Values
Age (y), mean (SD; range)		71 (4.8; 65 to 80)
**Sex, n (%)**		
	Male	8 (61.5)
	Female	5 (38.5)
**Race, n (%)**		
	Black or African American	1 (7.7)
	White	12 (92.3)
Ethnicity (not Hispanic or Latino), n (%)		13 (100)
**Marital status, n (%)**		
	Married	9 (69.2)
	Divorced or widowed	1 (7.7)
	Single	3 (23.1)
**Educational level, n (%)**		
	High school or lower	1 (7.7)
	Some college or post–high school training	4 (30.8)
	College graduate or higher	7 (53.8)
	Other or preferred not to say	1 (7.7)
**ECOG^a^** **performance status, n (%)**		
	0	1 (7.7)
	1	8 (61.5)
	2	4 (30.8)
**Diagnosis, n (%)**		
	Acute myeloid leukemia	7 (53.8)
	Chronic myelomonocytic leukemia	1 (7.7)
	Myelodysplastic syndrome	5 (38.5)
**Chemotherapy cycle at initiation of the intervention, n (%)**		
	1	3 (23.1)
	2	4 (30.8)
	3	3 (23.1)
	4	1 (7.7)
	>5	2 (15.4)
Daily steps at baseline, mean (SD; range)		3084 (1765.5; 649.8 to 6546.4)
Daily steps at the postintervention time point, mean (SD; range)		3757 (2623.6; 307.3 to 10,554.9)
Change in daily steps, mean (SD; range)		673 (1270.1; −1099.8 to 4008.4)
Frequency of resistance exercise (d per wk), mean (SD; range)		4 (2.2; 0.1 to 7.0)
Bout of resistance exercise (min), mean (SD; range)		25 (10.8; 8.3 to 46.1)
RPE^b^ during resistance exercise, mean (SD; range)		3 (1.3; 0.8 to 5.6)

^a^ECOG: Eastern Cooperative Oncology Group.

^b^RPE: rating of perceived exertion (1=“not tired at all”; 10=“extremely tired”).

### Change in Gait Outcomes From Baseline to the Postintervention Time Point

Table 3 describes gait outcomes at baseline and the postintervention time point and changes in these measures. At baseline, participants walked an average of 408.0 (SD 128.8) m in the 6-minute walk test, with a cadence of 110.9 (SD 12.4) steps per minute and a velocity of 82.0 (SD 21.8) meters per minute. The mean GSI was 95.1% (SD 3%). Stance duration accounted for an average of 59.9% (SD 1.9%) of the gait cycle, with a mean stance duration variability of 0.8% (SD 0.9%).

From baseline to the postintervention time point, we observed a numerical increase in average cadence (mean +4.6, SD 14.6 steps per minute; *P*=.24; ES=0.38) and velocity (mean +1.6, SD 8.2 meters per minute; *P*=.24; ES=0.07). We also observed a numerical increase in stance duration (mean +0.4%, SD 2.3%; *P*=.55; ES=0.20) and a numerical decrease in swing duration (mean −0.4%, SD 2.3%; *P*=.55; ES=0.20), which together brought stance and swing durations closer to their optimal ranges (Table 3). In terms of regularity outcomes, we observed a numerical decrease in variability for both normalized stride (mean −1.4%, SD 8.5%; *P*=.34; ES=−0.18) and swing duration (mean −0.1%, SD 1.1%; *P*=.54; ES=−0.15). None of these outcomes were statistically significant.

**Table 3:** Change in gait outcomes from baseline to the postintervention time point (n=13).

		Baseline		Postintervention time point		Change from baseline to postintervention time point			
		Mean (SD)	Median (IQR)	Mean (SD)	Median (IQR)	Mean (SD)	Median (IQR)	*P* value	Effect size
**Spatiotemporal outcomes**									
	6-min walking distance (meters)	408.0 (128.8)	434.1 (362.7 to 485.3)	411.9 (121.7)	449.6 (287.7 to 485.5)	+3.9 (74.5)	+15.5 (−14.7-28.2)	.85	0.03
	Cadence (steps per min)	110.9 (12.4)	113.0 (110.2 to 115.3)	115.5 (14.1)	115.5 (110.4 to 125.9)	+4.6 (14.6)	+2.0 (−0.8 to 5.0)	.24	0.38
	Velocity (meters per min)	82.0 (21.8)	84.6 (68.8 to 94.6)	83.5 (24.7)	86.4 (76.3 to 97.0)	+1.6 (8.2)	+3.7 (−1.6 to 6.1)	.24	0.07
	Stride length (meters)	1.5 (0.3)	1.6 (1.2 to 1.7)	1.4 (0.3)	1.5 (1.3 to 1.7)	−0.1 (0.2)	−0.0 (−0.1 to 0.0)	.54	−0.16
	Stride length (% of height)	89.1 (19.2)	93.4 (73.6 to 102.0)	86.0 (19.2)	90.4 (73.6 to 98.2)	−3.1 (10.8)	−0.1 (−5.1 to 1.2)	.54	−0.16
	General symmetry index (%)	95.1 (3)	95.7 (92.9 to 98.0)	95.1 (2.4)	95.8 (94.2 to 96.4)	−0.0 (2.1)	−0.8 (−1.2 to 1.8)	.99	−0.00
	Swing duration (% of cycle)	40.1 (1.9)	40.2 (39.4 to 41.5)	39.7 (2.5)	40.2 (37.1 to 41.6)	−0.4 (2.3)	−0.2 (−1.2 to 0.9)	.55	−0.20
	Stance duration (% of cycle)	59.9 (1.9)	59.8 (58.5 to 60.6)	60.3 (2.5)	59.8 (58.4 to 62.9)	+0.4 (2.3)	+0.2 (−0.9 to 1.2)	.55	0.20
**Regularity outcomes**									
	Stride length variability (% of height)	5.9 (7.6)	4.1 (2.6 to 5.5)	4.5 (2.8)	3.3 (3.0 to 4.5)	−1.4 (8.5)	+0.8 (0.2 to 1.1)	.34	−0.18
	Swing duration variability (% of cycle)	0.8 (0.9)	0.5 (0.4 to 0.8)	0.7 (0.6)	0.5 (0.4 to 0.6)	−0.1 (1.1)	−0.1 (−0.3 to 0.2)	.54	−0.15

### Correlations Between Both Change in Daily Steps and Resistance Exercise Duration and Changes in Gait Outcomes

Table 4 presents the correlations between changes in daily steps and duration of resistance band exercise with changes in gait outcomes. Increased daily steps significantly correlated with decreased swing duration variability (*r*=−0.72; *P*=.01). Duration of resistance band exercise significantly correlated with increased distance covered in the 6-minute walk test (*r*=0.62; *P*=.03), cadence (*r*=0.54; *P*=.06), velocity (*r*=0.56; *P*=.05), and stance duration (*r*=0.58; *P*=.04). We did not observe significant correlations for other gait outcomes.

Scatterplots showing the relationship between changes in gait outcomes and changes in exercise are shown in Figures S1 and S2 in Multimedia Appendix 3. In sensitivity analysis excluding one potential influential observation (Table S2 in Multimedia Appendix 3), the correlations remained largely consistent. Increased daily steps continued to be correlated with reduced swing duration variability (*r*=−0.64; *P*=.02). Similarly, longer resistance band exercise duration was correlated with improved walking distance (*r*=0.59; *P*=.04) and velocity (*r*=0.54; *P*=.07). Correlations of resistance band exercise duration with cadence (*r*=0.45; *P*=.14) and stance duration (*r*=0.50; *P*=.10) remained in the same direction.

**Table 4:** Correlations between change in gait outcomes and change in exercise (n=13).

Gait parameter		Change in daily steps		Duration of resistance training (min)	
		*r*	*P* value	*r*	*P* value
**Change in spatiotemporal outcomes**					
	6-min walking distance (meters)	0.16	.59	0.62	.03
	Cadence (steps per min)	0.20	.51	0.54	.06
	Velocity (meters per min)	0.24	.43	0.56	.05
	Stride length (meters)	0.05	.87	0.03	.93
	Stride length (% of height)	0.05	.87	0.03	.93
	General symmetry index (%)	0.10	.75	0.19	.54
	Swing duration (% of cycle)	0.26	.38	−0.58	.04
	Stance duration (% of cycle)	−0.26	.38	0.58	.04
**Change in regularity outcomes**					
	Stride length variability (% of height)	−0.24	.43	−0.15	.62
	Swing duration variability (% of cycle)	−0.72	.01	0.10	.75

## Discussion

In this pilot mobile health exercise study for older adults with MNs, we observed numerical changes in several gait outcomes, including cadence (mean +4.6, SD 14.6 steps per minute; *P*=.24; ES=0.38), stance duration (mean +0.4%, SD 2.3%; *P*=.55; ES=0.20), swing duration (mean −0.4%, SD 2.3%; *P*=.55; ES=0.20), and variability in normalized stride (mean −1.4%, SD 8.5%; *P*=.34; ES=−0.18) and swing duration (mean −0.1%, SD 1.1%; *P*=.54; ES=−0.15). Better intervention adherence significantly correlated with improved gait outcomes. These findings suggest that adherence to the GO-EXCAP mobile health exercise intervention shows promise in improving gait in this population.

To our knowledge, no studies have evaluated gait outcomes following exercise interventions in older adults with MNs. Therefore, we compare our results to those of previous research on older adults with other types of cancer. In a 12-week program incorporating electromyostimulation, light-intensity exercise, and dietary support for patients with advanced-stage solid cancers (mean age 62.4 years), participants in the intervention arm (n=26) showed improvements in mean cadence (+4.2 steps per minute) and stance duration (0.5% closer to the optimal range) [20]. In another 8-week home-based resistance training for older patients with advanced cancer (n=30; median age 75 years), mean gait velocity increased by 2.4 meters per minute among those with ≥80% intervention compliance [21]. While these interventions differ from ours in exercise type and duration, the favorable changes in gait outcomes observed are comparable. Taken together, these studies suggest that exercise interventions, including GO-EXCAP, have the potential to address spatiotemporal outcomes in older adults with cancer and need to be studied further.

In addition to commonly analyzed spatiotemporal outcomes, our study expands the literature by evaluating gait symmetry in older adults with MNs. Gait asymmetries are clinically relevant as they have been linked to a high risk of fall [22,23]. There are several likely explanations for the lack of significant change in symmetry in our sample. First, the GSI reported in our study (95.1%) is slightly higher than that reported for healthy adults aged 61 years and older (92%-94%) [24]. Although there is a lack of normative data for this measure, this value suggests that our study sample may have preserved physical function, reflected in a relatively good gait symmetry at baseline, leaving limited room for improvement. In addition, symmetry can be assessed in different movement directions, such as anterior or posterior (evaluated in our study) and medial or lateral. Several studies have linked pathological gait patterns to a higher risk of falls in the medial or lateral direction, suggesting that non–anterior or posterior assessments may provide more valuable insights into gait symmetry [4]. Future studies are warranted to determine which gait symmetry measures are most sensitive in detecting clinically relevant pathological gait outcomes.

Adherence to GO-EXCAP shows promise in improving the variability of stride and swing duration, which quantifies the automaticity and regularity of gait, among older patients with MNs. Increased gait variability is associated with a higher risk of falls, frailty, and incident mobility disability [25-27]. Notably, gait variability may be a more sensitive marker than spatiotemporal outcomes (eg, gait velocity) for detecting pathological gait in older adults with cancer who report peripheral neuropathy [7]. Despite its predictive values, we found only one previous study that evaluated gait variability following an exercise intervention, and the authors reported no significant changes [20]. Thus, our study expands the literature by demonstrating a statistically significant correlation between adherence to the GO-EXCAP intervention and improved gait regularity in older adults with MNs. Increased exercise, including progressive aerobic walking, may improve gait regularity through enhancing motor control and gait automaticity [28]. More studies are needed to better understand these underlying mechanisms.

The strengths of this study include the use of a user-friendly and noninvasive motion sensor to measure gait outcomes, which allowed for efficient gait dataset management, and the comprehensive measurement of gait by including both spatiotemporal and regularity outcomes. Several limitations should also be noted. First, given the pilot nature of this study, the correlation analyses were exploratory and hypothesis generating. Second, the single-arm design without a control group prevents us from determining whether observed gait improvements are attributed directly to the intervention. Third, the small sample size limited our ability to adjust for potential confounders or provide CI estimates for correlation analysis. Additionally, to our knowledge, established minimal clinically important difference thresholds for gait outcomes are not available for general older adults or for older adults with cancer. Therefore, it remains unclear whether the observed improvements in gait outcomes reached a clinically meaningful threshold. Finally, there is no consensus on clinically meaningful differences in gait spatiotemporality and regularity outcomes. Therefore, our correlation analysis between change in gait outcomes and change in exercise should be interpreted as exploratory and is primarily useful to guide future larger analyses of similar outcomes in this population.

In conclusion, we found that greater exercise adherence to GO-EXCAP significantly correlated with improved gait outcomes, suggesting that the combination of aerobic and resistance training has the potential to improve gait quality in inactive older adults with cancer. Our ongoing pilot randomized controlled trial (ClinicalTrials.gov identifier: NCT04981821) will further examine the effects of the GO-EXCAP intervention on gait outcomes in this population and assess whether improvements in gait are associated with better functional outcomes, including reduced falls.

## Appendix

Multimedia Appendix 1 TREND checklist.

Multimedia Appendix 2 Strategy used to determine the weekly progression rate.

Multimedia Appendix 3 Baseline characteristic comparisons between participants, scatterplots showing the relationship between changes in gait outcomes and changes in exercise, and sensitivity analysis.

## Abbreviations

AML: acute myeloid leukemia
ECOG: Eastern Cooperative Oncology Group
ES: effect size
EXCAP: Exercise for Cancer Patients
GO-EXCAP: Geriatric Oncology-Exercise for Cancer Patients
GSI: general symmetry index
MDS: myelodysplastic syndrome
MN: myeloid neoplasm
REDCap: Research Electronic Data Capture
RPE: rating of perceived exertion
TREND: Transparent Reporting of Evaluations With Nonrandomized Designs

## Data Availability

The datasets generated or analyzed during this study are available from the corresponding author on reasonable request.
