# Supplementary material for: Gait Changes After a Mobile Health Exercise Intervention in Older Adults With Myeloid Neoplasms: Single-Arm Pilot Trial
Source: JMIR Cancer. 2026 Apr 29;12:e80909. doi: 10.2196/80909 (PMC13173077; doi:10.2196/80909)
Supplement: Multimedia Appendix 2 [file cancer_v12i1e80909_app2.docx]

**Criteria and strategy for determining weekly step goal progression rate (5%-20%)**

The 5%-20% step progression prescription was individualized based on three key criteria: 1) averaged daily step counts at baseline, 2) participants’ physical capability and perceived readiness, and 3) weekly performance during the intervention. A trained ACSM-certified clinical exercise physiologist prescribed the individualized walking step progression based on each participant’s average daily steps at baseline, with the goal of progressing toward approximately 10,000 steps per day by the end of the 12-week GO-EXCAP intervention period. Specifically, participants with lower baseline step counts were prescribed a higher weekly progression rate (up to 20%), whereas those with higher baseline step counts received lower weekly progression rates.

In addition to baseline step counts, the clinical exercise physiologist considered each participant’s physical capacity and perceived readiness when determining the weekly progression rate. This individualized approach ensured that the weekly step goals were appropriate, achievable, and aligned with the participant’s physical capacity, thereby minimizing the risk of nonadherence associated with overly aggressive goals.

Throughout the intervention period, the clinical exercise physiologist logged into the web portal at least twice weekly to monitor intervention adherence, provide feedback, and modify exercise prescriptions as needed. If a participant met or exceeded their weekly step goal, the progress rate for the subsequent week may be maintained or increased (up to a maximum of 20%). Conversely, if a participant had difficulty meeting their prescribed step goal, the clinical exercise physiologist would collaborate with the participant to identify barriers, provide solutions, and modify the progression rate to enhance intervention adherence.

Overall, the individualized step progression was determined by baseline step counts, physical capability, and weekly performance, with weekly monitoring and adjustments made as needed throughout the intervention period.

For progressive aerobic walking, walking speed (e.g., target pace, cadence, or intensity) was not measured. Instead, adherence to aerobic walking was quantified using daily step counts collected via the GO-EXCAP application.
