# Supplementary material for: Gait Changes After a Mobile Health Exercise Intervention in Older Adults With Myeloid Neoplasms: Single-Arm Pilot Trial
Source: JMIR Cancer. 2026 Apr 29;12:e80909. doi: 10.2196/80909 (PMC13173077; doi:10.2196/80909)

Table S1. Comparison of demographics and clinical characteristics between patients included in the analysis and those who were not

| Variable | Included (n=13) | Excluded (n=12) | p |
| --- | --- | --- | --- |
| Age in years, mean (SD, range) | 71 (4.8, 65-80) | 73 (5.1, 62.0-81.0) | 0.56 |
| Sex, n (%) |  |  | 0.67 |
| Male | 8 (61.5) | 9 (75.0) |  |
| Female | 5 (38.5) | 3 (25.0) |  |
| Race, n (%) |  |  | 0.37 |
| Black or African American | 1 (7.7) | - |  |
| White | 12 (92.3) | 11 (91.7) |  |
| Prefer not to say | - | 1 (8.3) |  |
| Ethnicity, n (%): |  |  | 0.48 |
| Not Hispanic or Latino | 13 (100.0) | 11 (91.7) |  |
| Prefer not to say | - | 1 (8.3) |  |
| Marital status, n (%) |  |  | 0.49 |
| Married | 9 (69.2) | 7 (58.3) |  |
| Divorced or widowed | 1 (7.7) | 3 (25.0) |  |
| Single | 3 (23.1) | 2 (16.7) |  |
| Education, n (%) |  |  | 0.57 |
| High school or less | 1 (7.7) | 3 (25.0) |  |
| Some college/post-high school training | 4 (30.8) | 2 (16.7) |  |
| College graduate or above | 7 (53.8) | 7 (58.3) |  |
| Other/prefer not to say | 1 (7.7) | - |  |
| Eastern Cooperate Group (ECOG) performance status, n (%) |  |  | 0.43 |
| 0 | 1 (7.7) | 3 (25.0) |  |
| 1 | 8 (61.5) | 7 (58.3) |  |
| 2 | 4 (30.8) | 2 (16.7) |  |
| Diagnosis, n (%) |  |  | 0.47 |
| Acute myeloid leukemia | 7 (53.8) | 8 (66.7) |  |
| Chronic myelomonocytic leukemia | 1 (7.7) | - |  |
| Chronic neutrophilic leukemia | - | 1 (8.3) |  |
| Myelodysplastic syndrome | 5 (38.5) | 3 (25.0) |  |
| Chemotherapy cycle at initiation of intervention, n (%)* |  |  | 0.14 |
| 1 | 3 (23.1) | - |  |
| 2 | 4 (30.8) | 9 (75.0) |  |
| 3 | 3 (23.1) | 1 (8.3) |  |
| 4 | 1 (7.7) | - |  |
| >5 | 2 (15.4) | 1 (8.3) |  |
| Average daily steps at baseline, mean (SD, range) | 3084 (1765.5, 649.8-6546.4) | 2649 (2187.6, 480.7-8756.8) | 0.42 |
| P values were generated from the hypotheses that test differences in demographics and clinical characteristics between patients included in the analysis and those who were not.  SD: standard deviation; RPE: rating of perceived exertion.  *For one patient excluded from the analysis, hypomethylating agent was planned but switched to hydroxyurea following enrollment. | | | |

Table S2. Sensitivity analysis excluding potential influential observations: correlations between change in gait outcomes and change in exercise

| Gait parameters | Change in steps | Duration of resistance training |
| --- | --- | --- |
| **Change in spatio-temporal outcomes** | |  |
| Walking distance (meters) | r=0.05; p=0.88 n=12 | r=0.59; p=0.04 n=12 |
| Cadence (steps/min) | r=0.06; p=0.86 n=12 | r=0.45; p=0.14 n=12 |
| Velocity (meters/minute) | r=0.15; p=0.65 n=12 | r=0.54; p=0.07 n=12 |
| Stride length (meters) | r=0.34; p=0.29 n=12 | r=0.22; p=0.50 n=12 |
| % Stride length (% height) | r=0.34; p=0.29 n=12 | r=0.22; p=0.50 n=12 |
| General Symmetry Index | r=-0.15; p=0.65 n=12 | r=0.03; p=0.91 n=12 |
| Swing duration (% cycle) | r=0.61; p=0.04 n=12 | r=-0.50; p=0.10 n=12 |
| Stance Duration (% cycle) | r=-0.61; p=0.04 n=12 | r=0.50; p=0.10 n=12 |
| **Change in regularity outcomes** | |  |
| % Stride length (% height) variability | r=-0.03; p=0.91 n=12 | r=-0.04; p=0.90 n=12 |
| Swing Duration (% cycle) variability | r=-0.64; p=0.02 n=12 | r=0.27; p=0.39 n=12 |
| In this sensitivity analysis, we repeated Spearman’s correlation analyses after excluding one observation that was more than 1.5 interquartile range below the first quartile and did not follow the general trend of the rest of the data. | | |

Figure S1. Scatter plots showing the relationship between change in gait outcomes and change in average daily steps from baseline to post-intervention


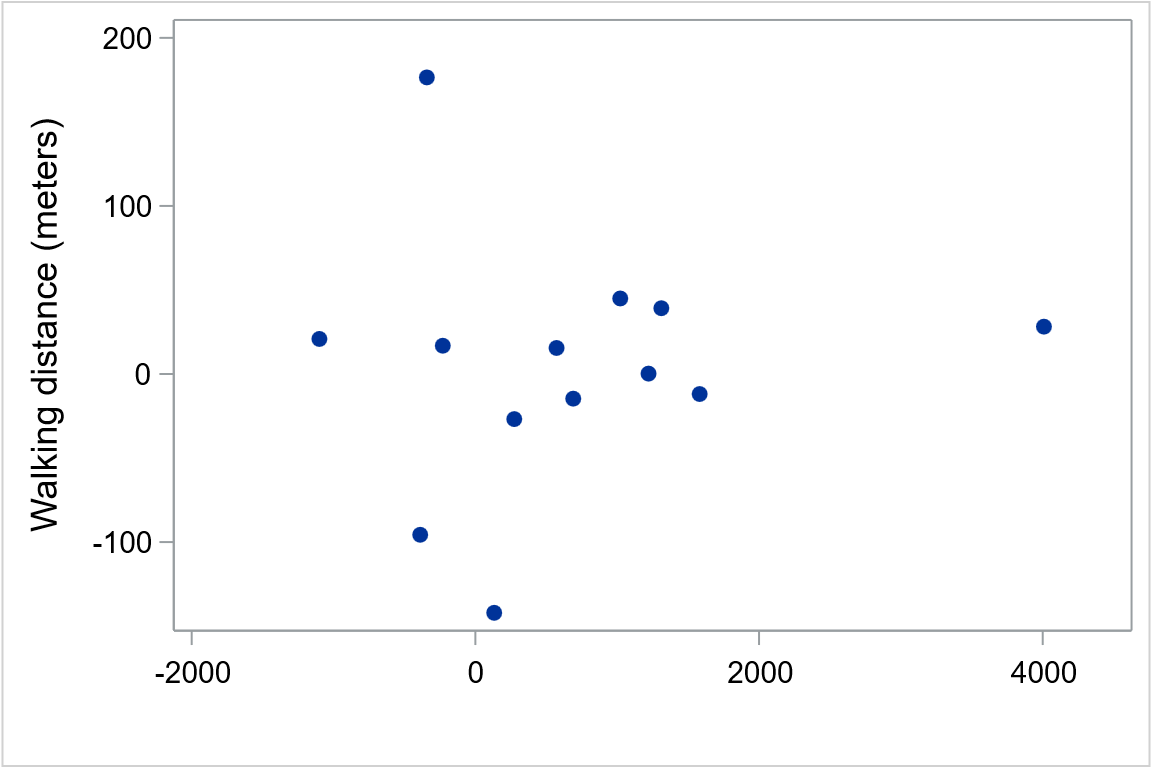

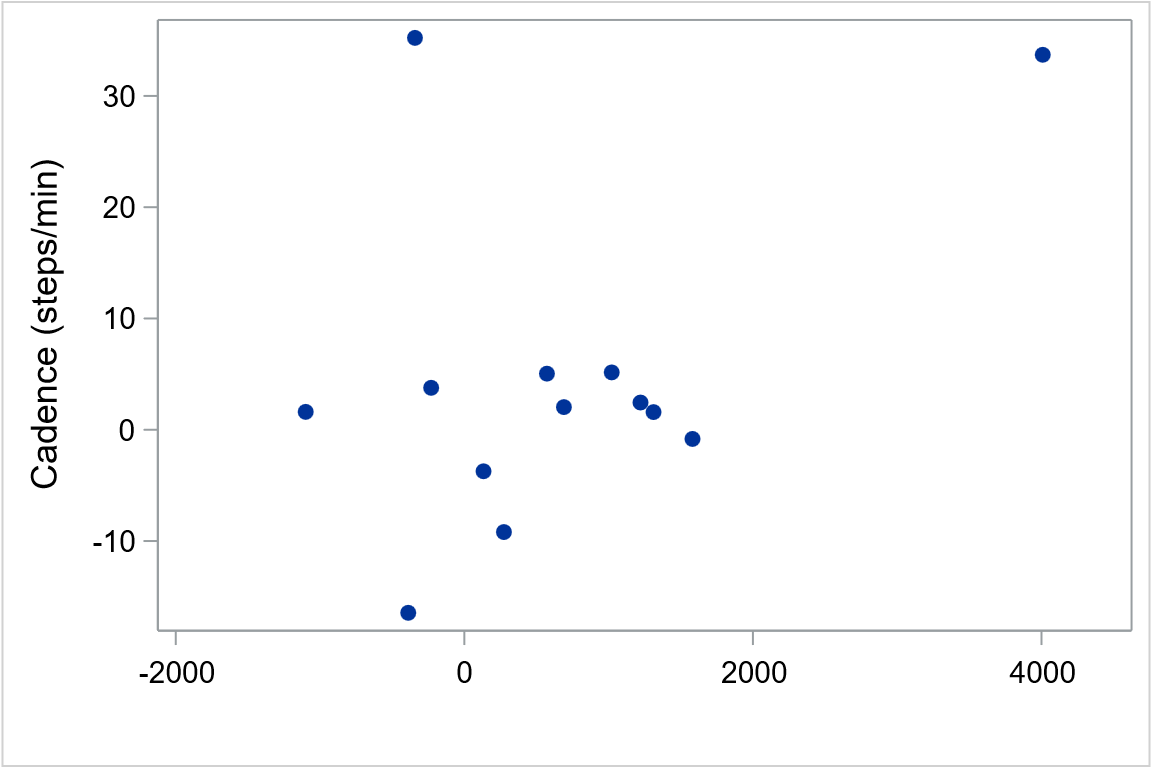

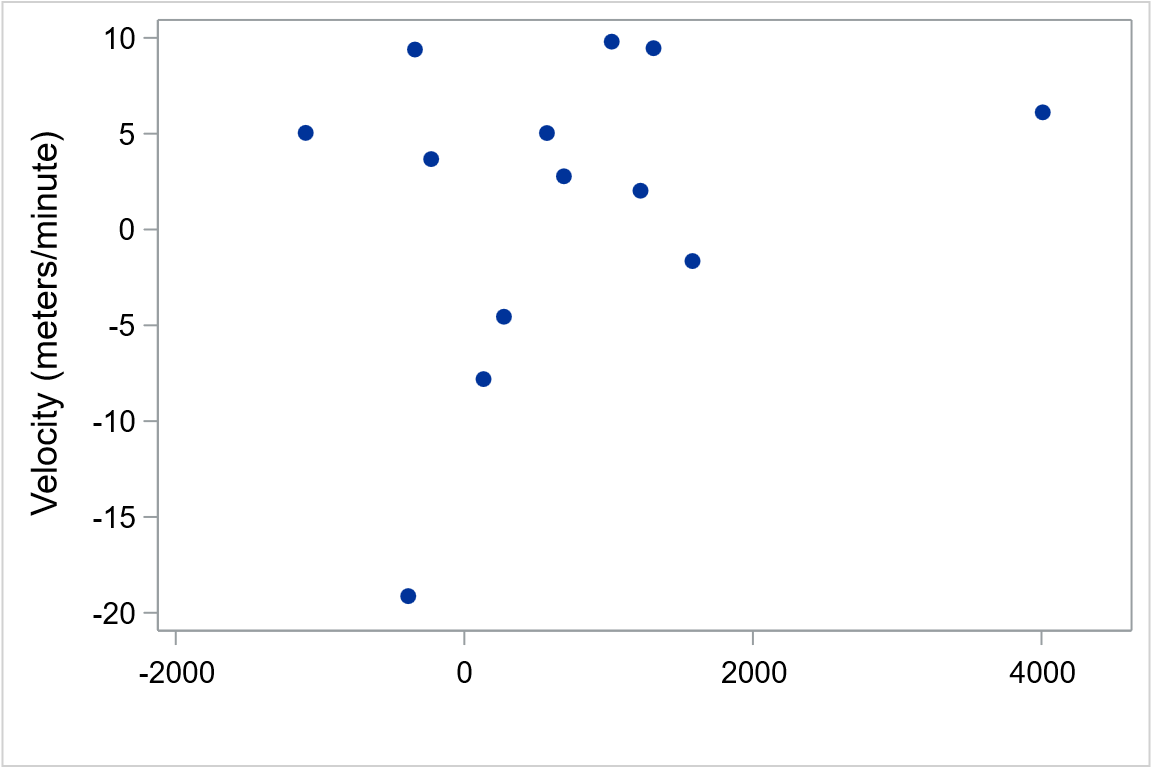

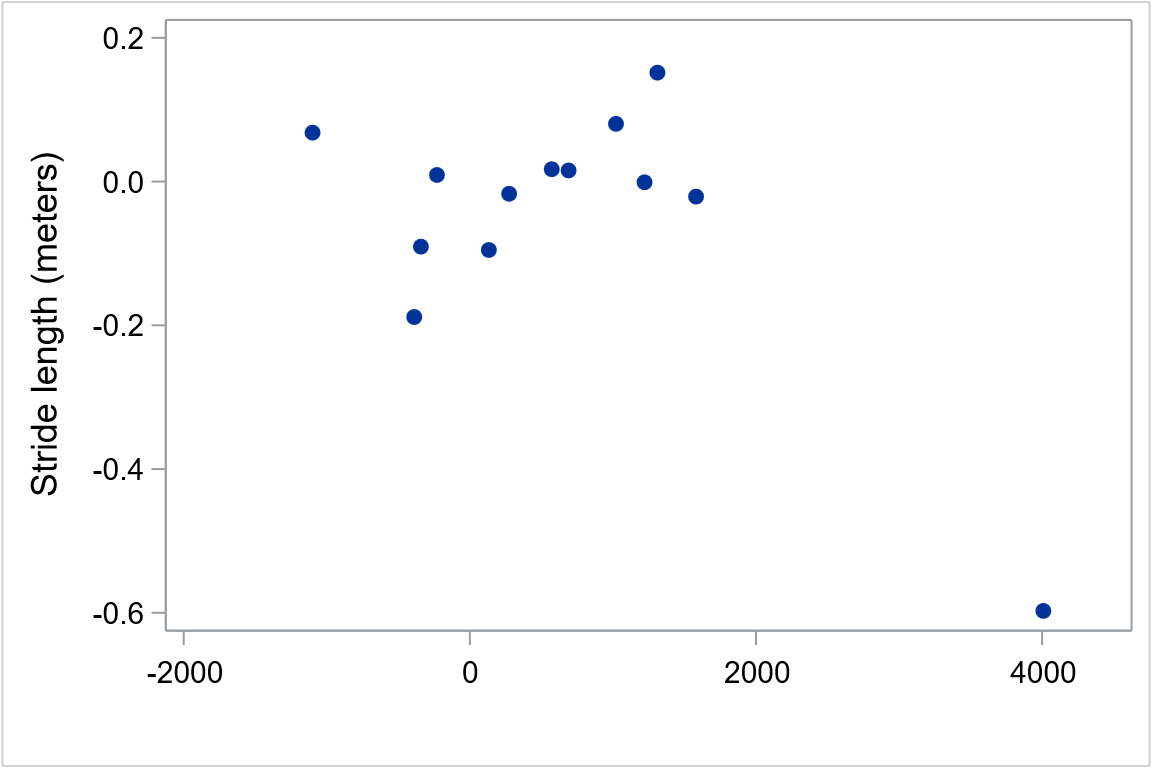

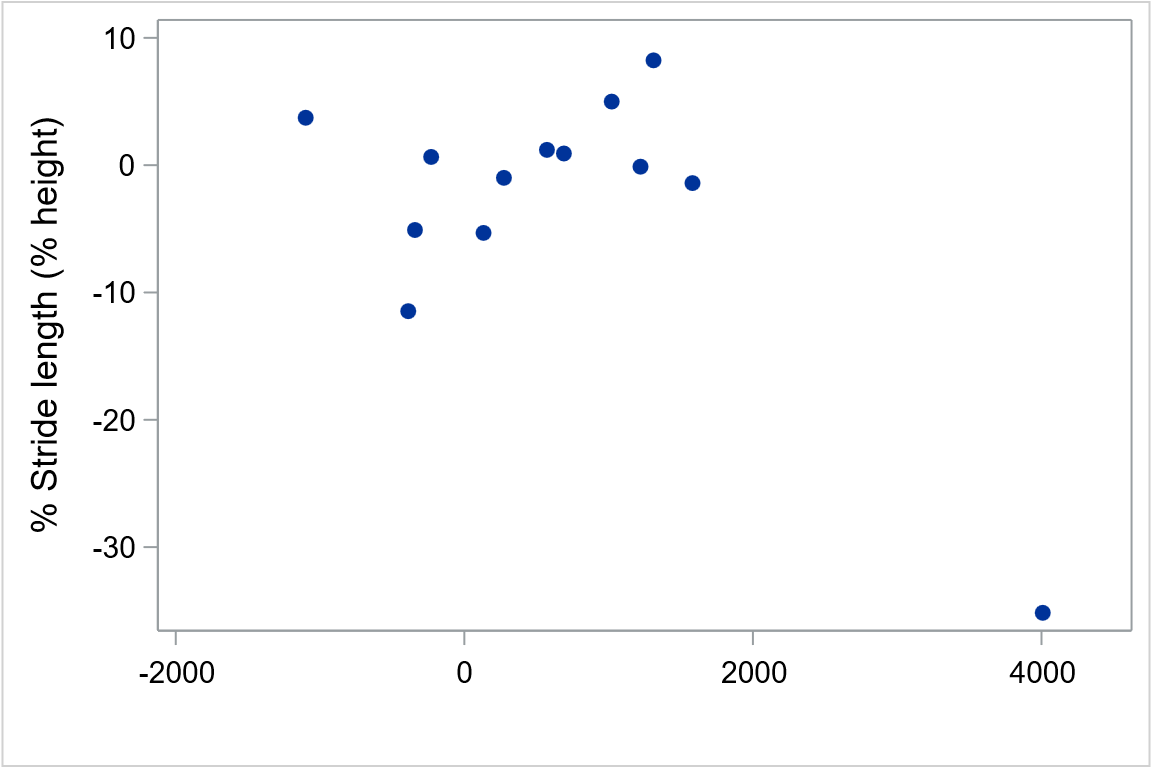

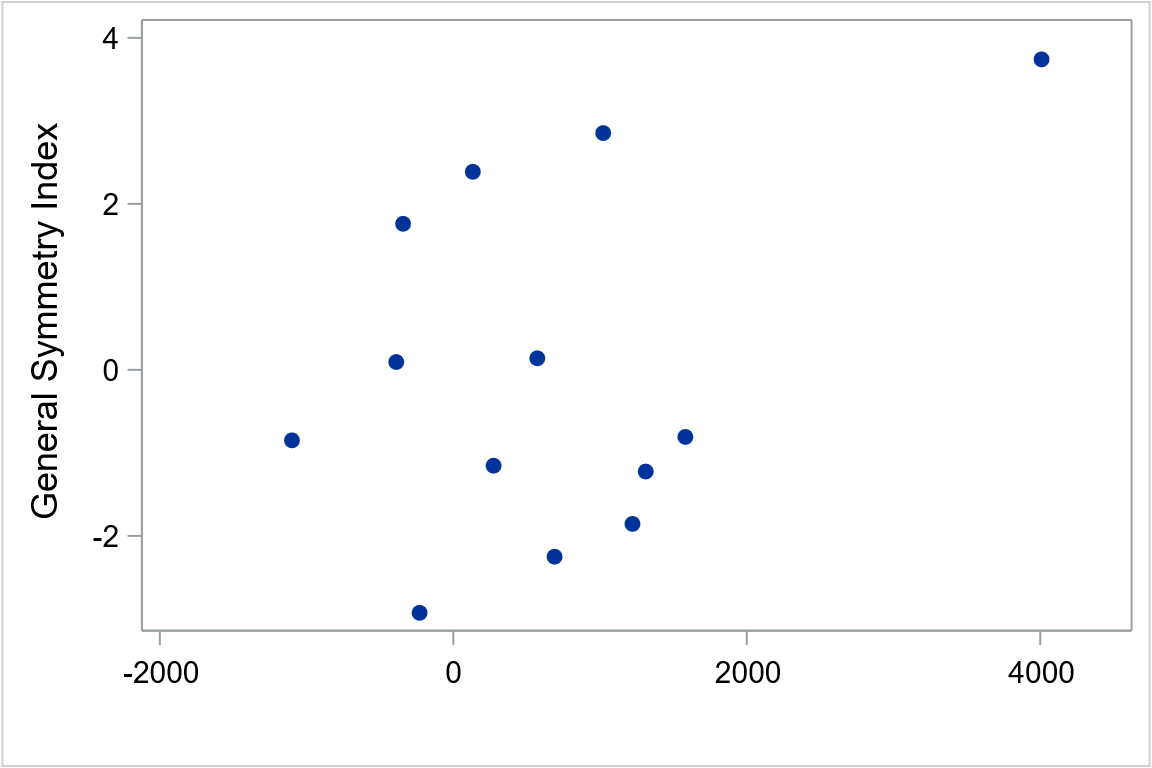

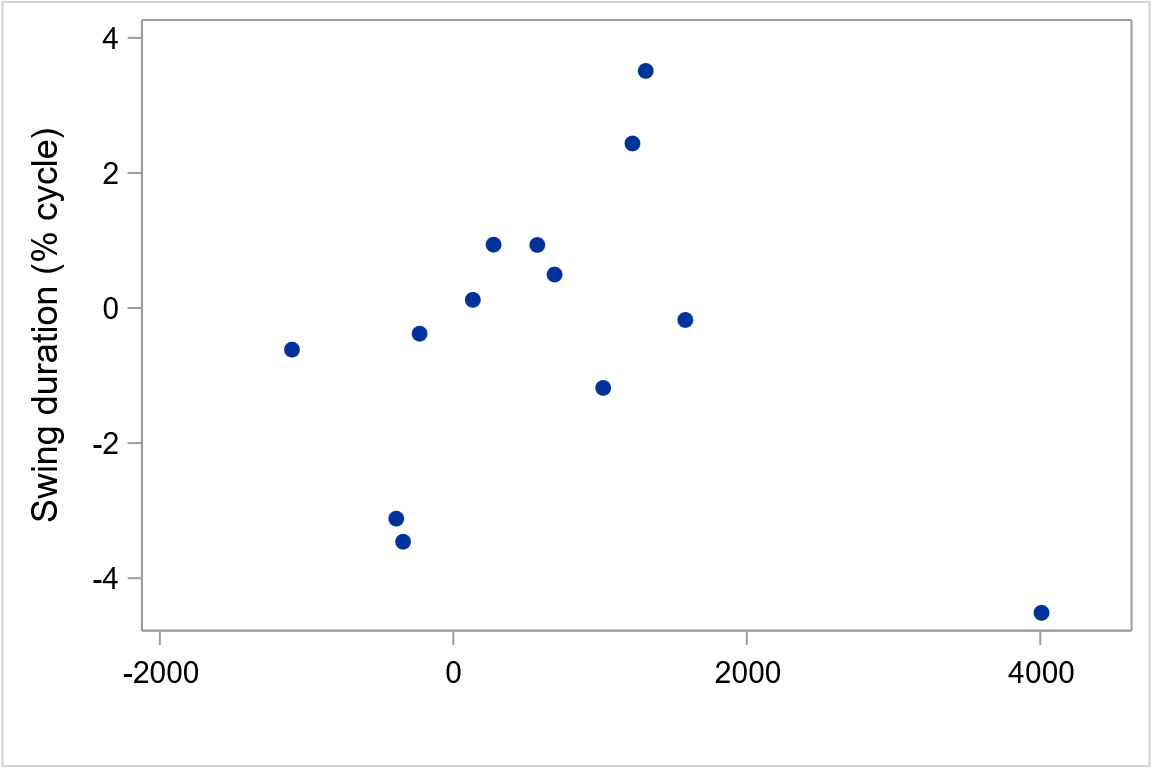

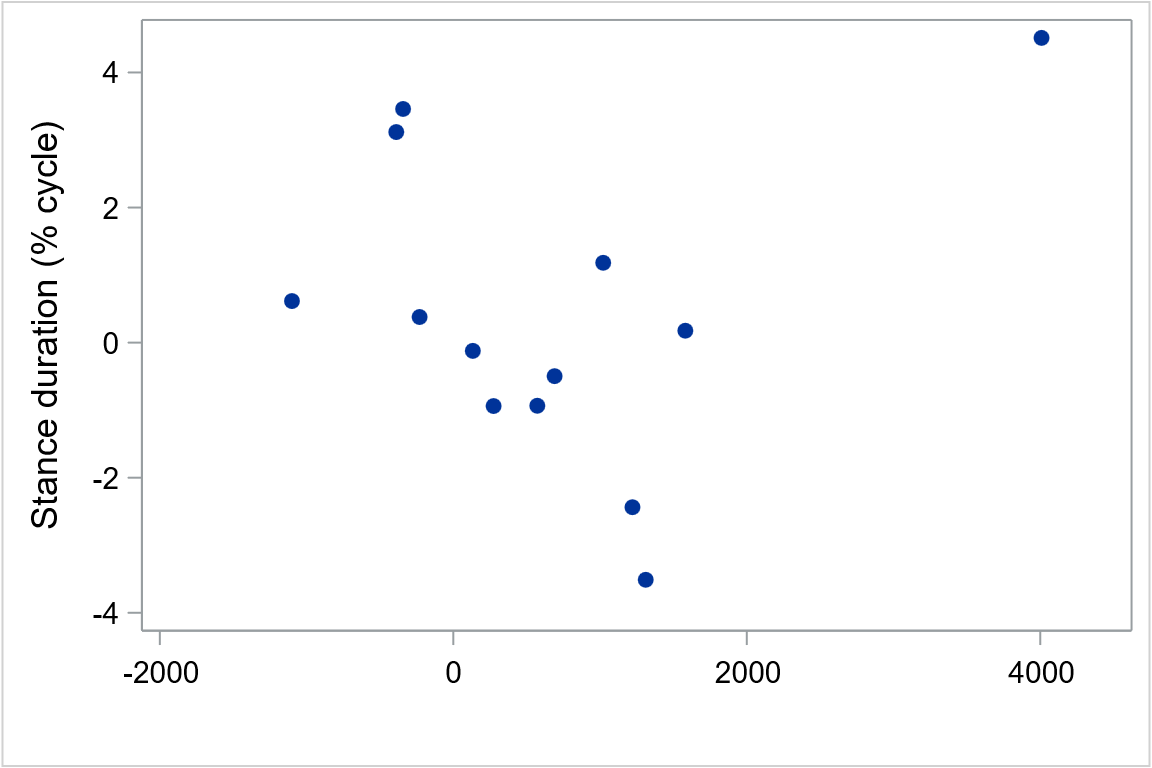

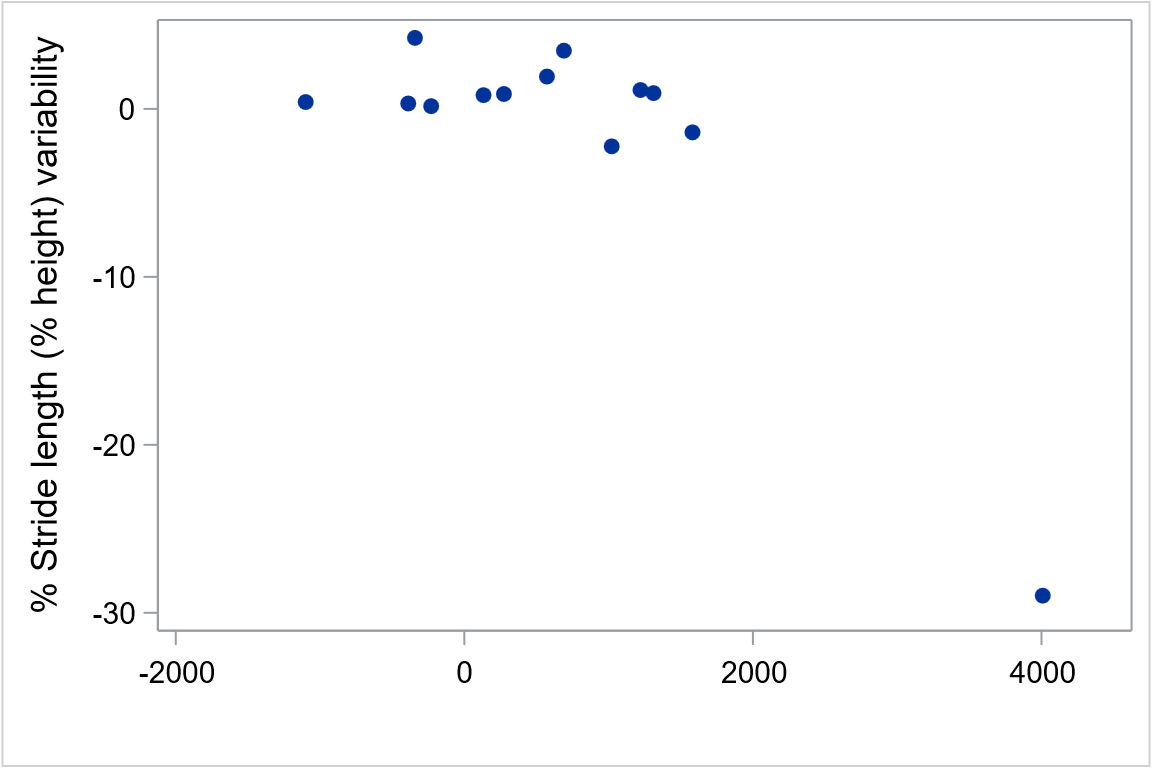

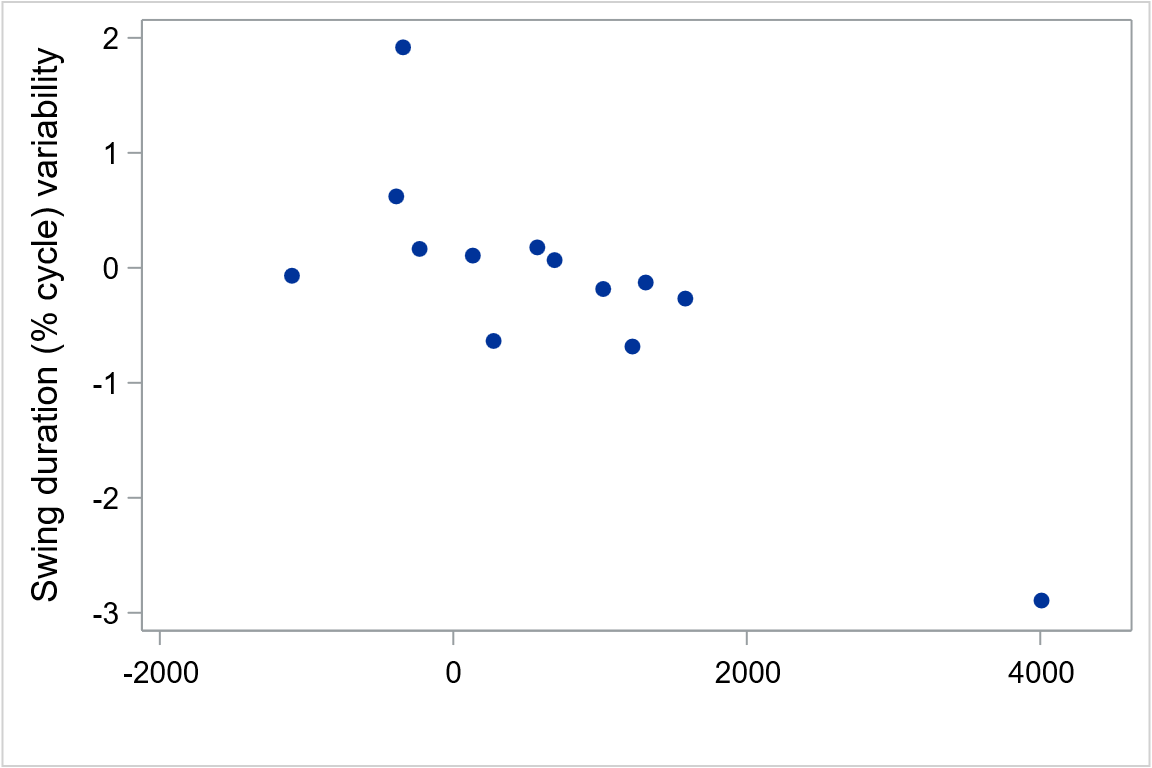


Figure S2. Scatter plots showing the relationship between change in gait outcomes and average daily minutes of resistance exercise


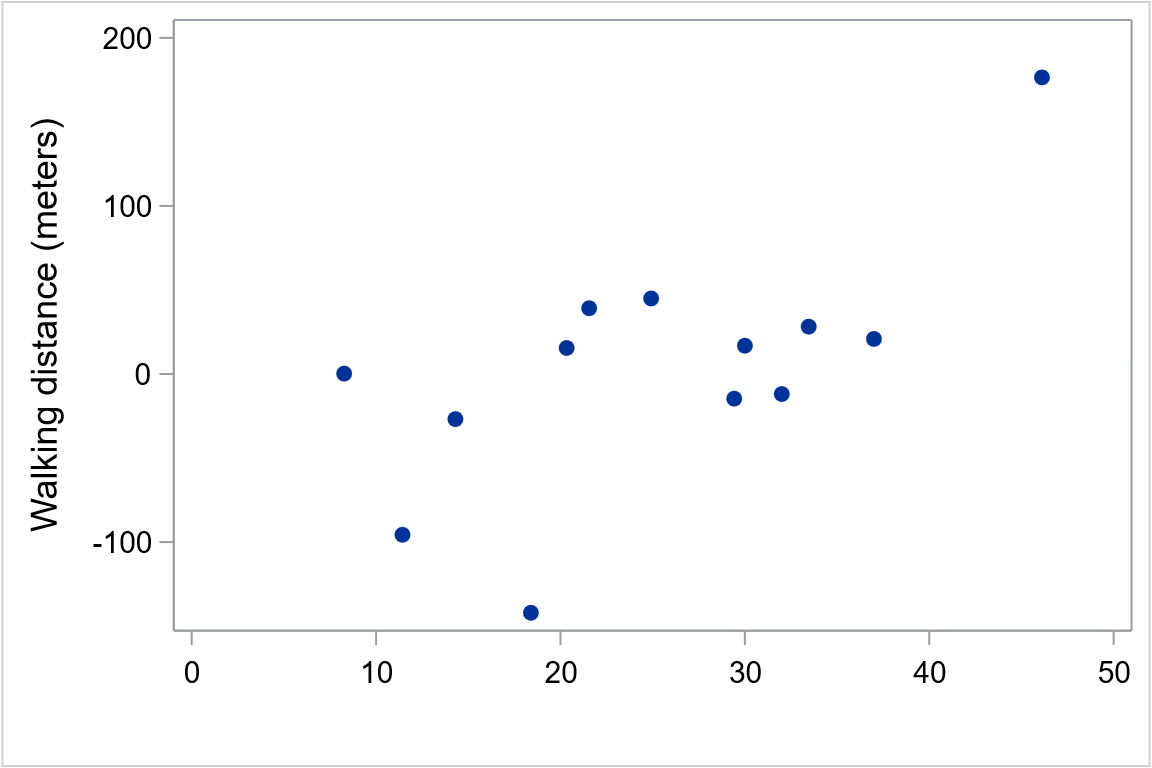

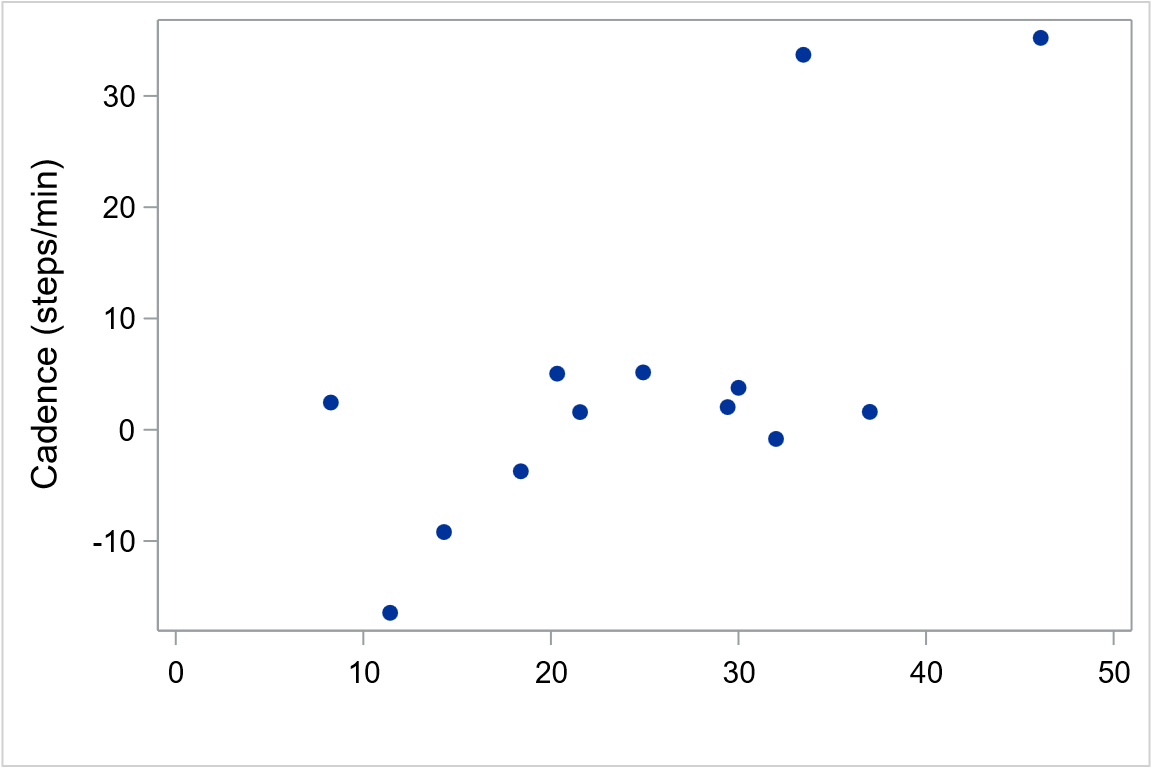

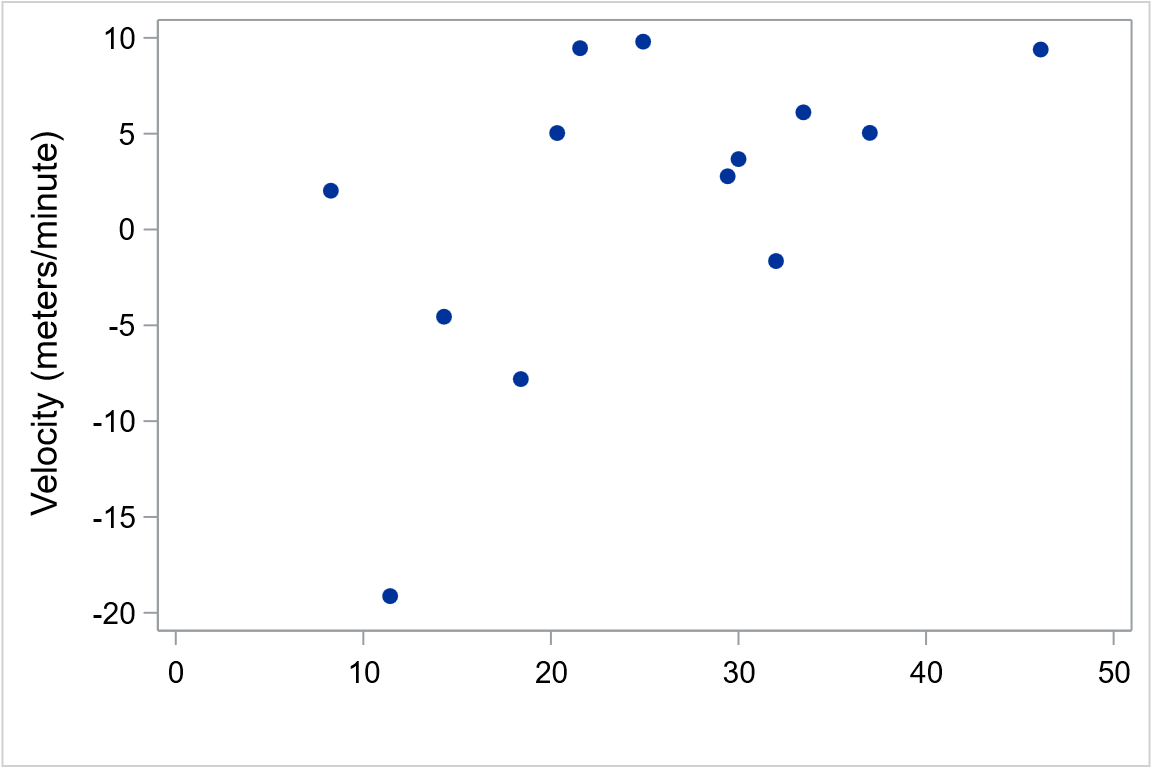

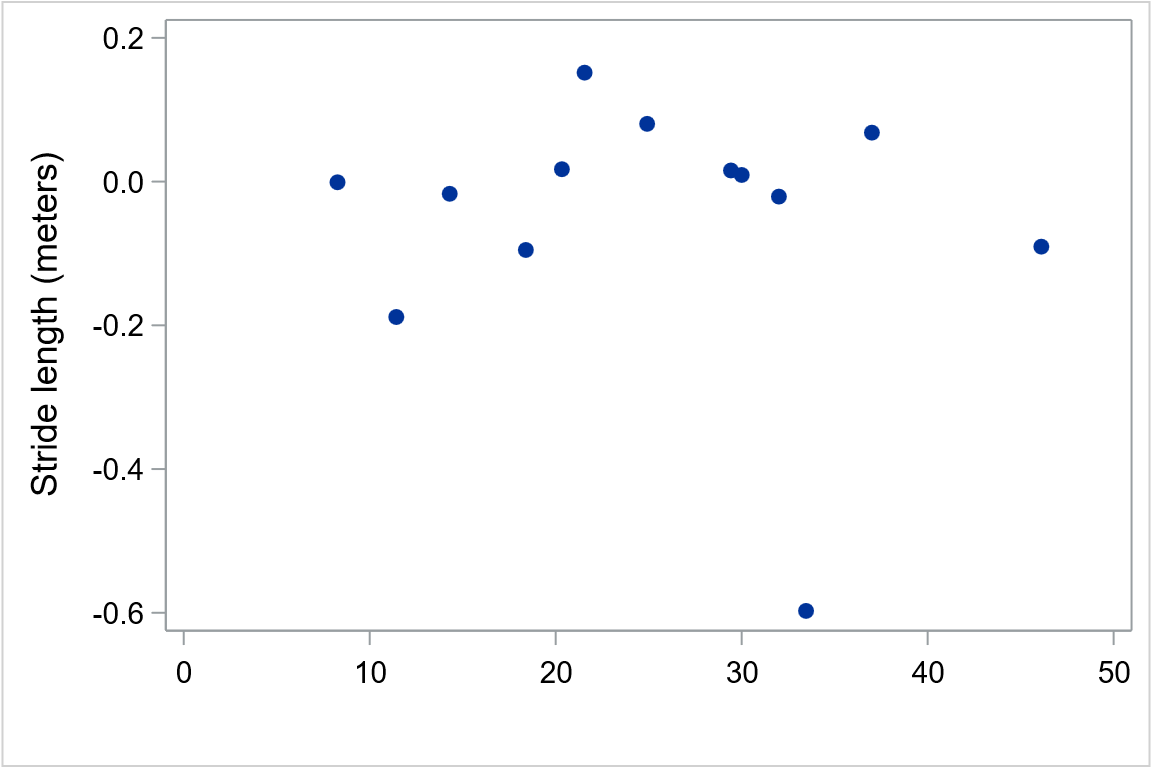

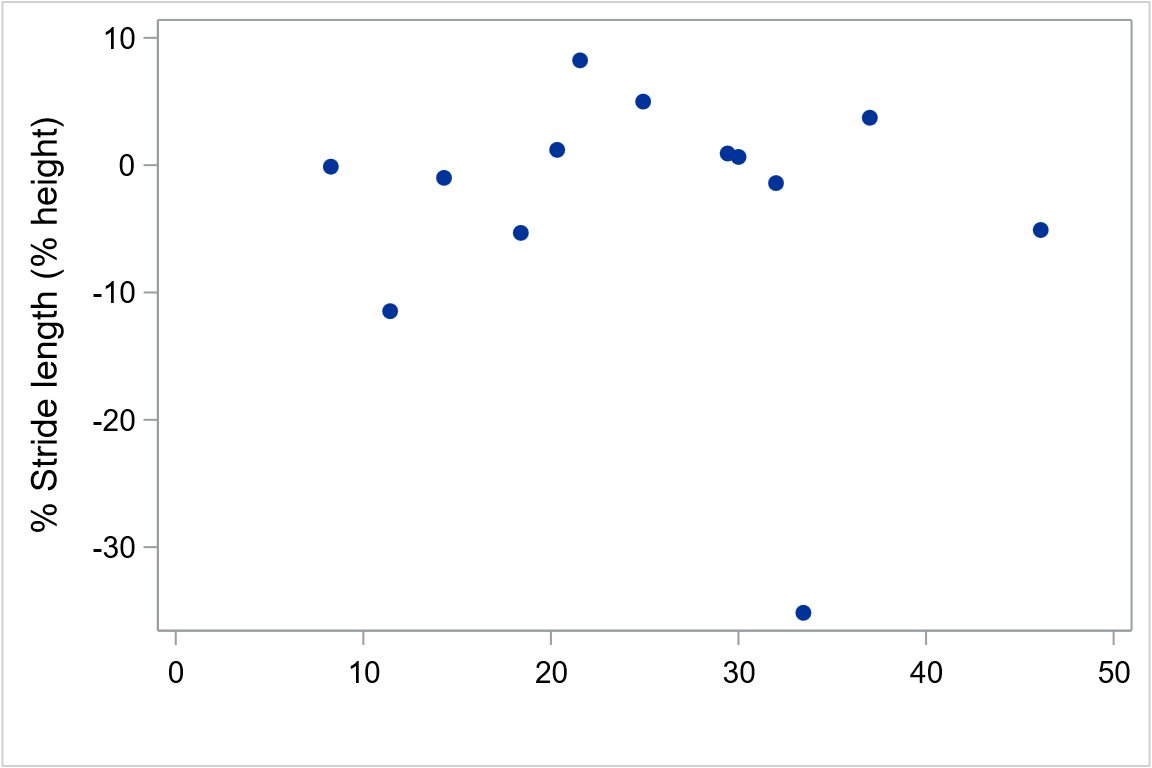

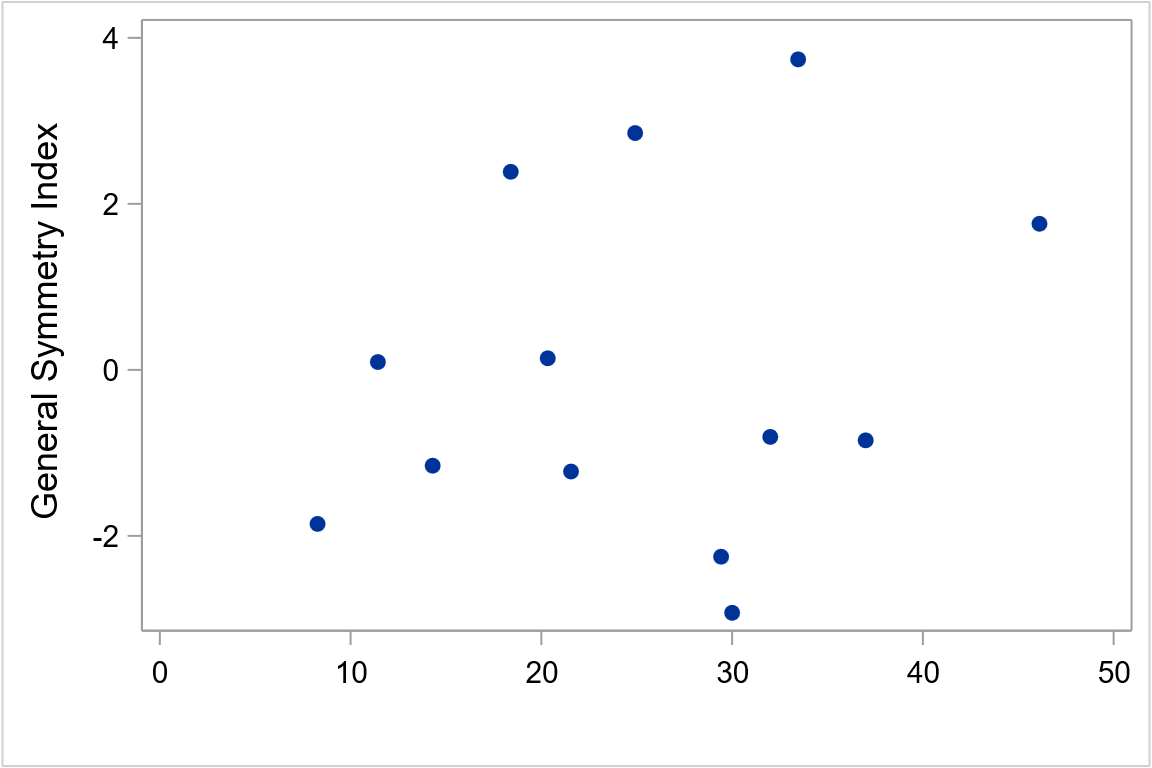

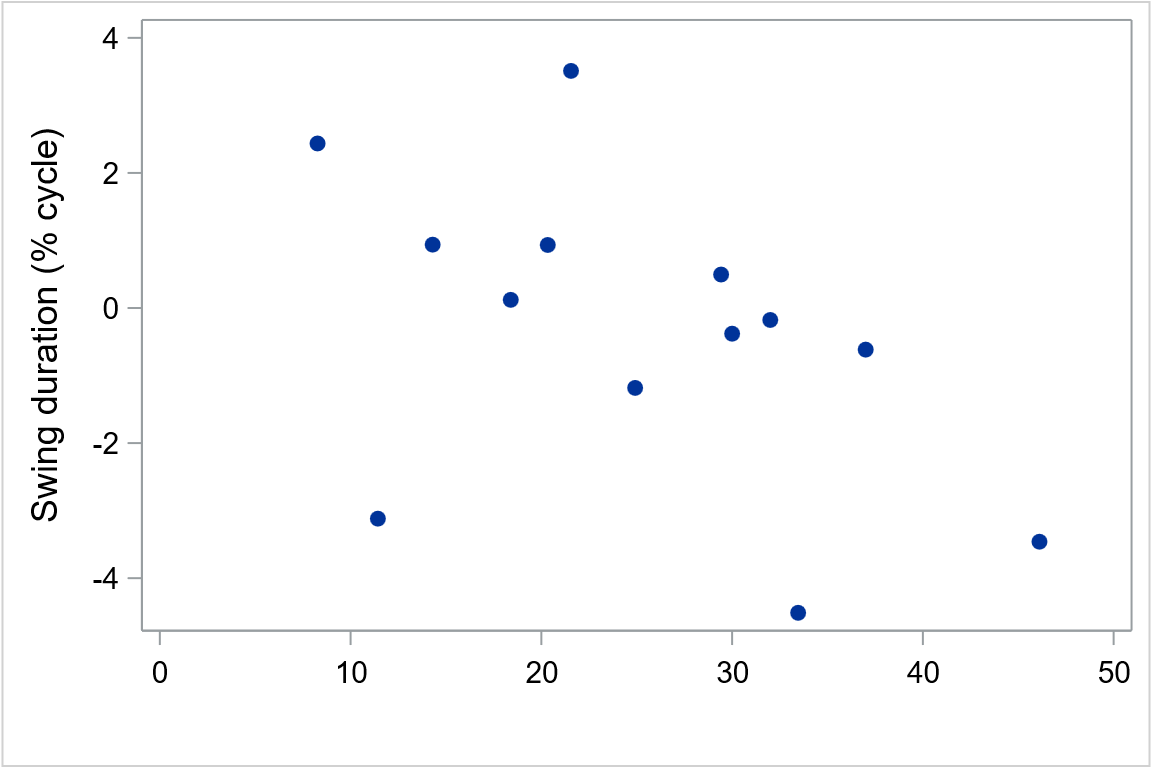

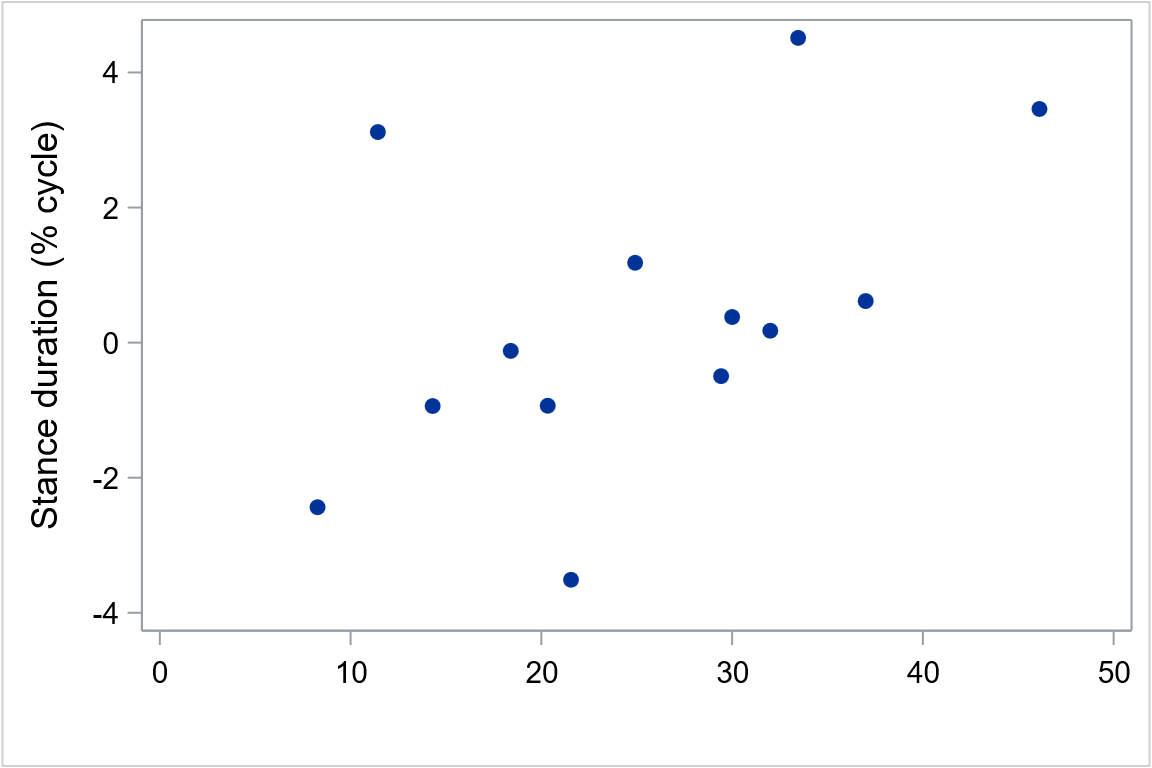

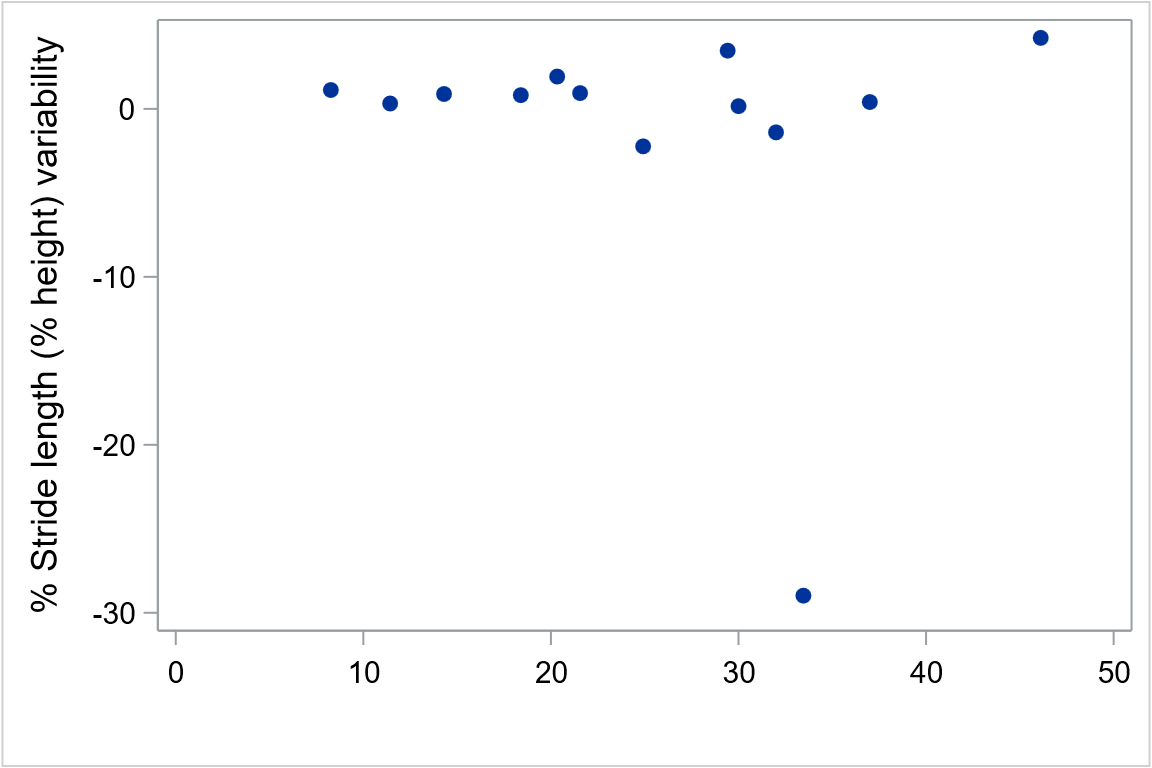

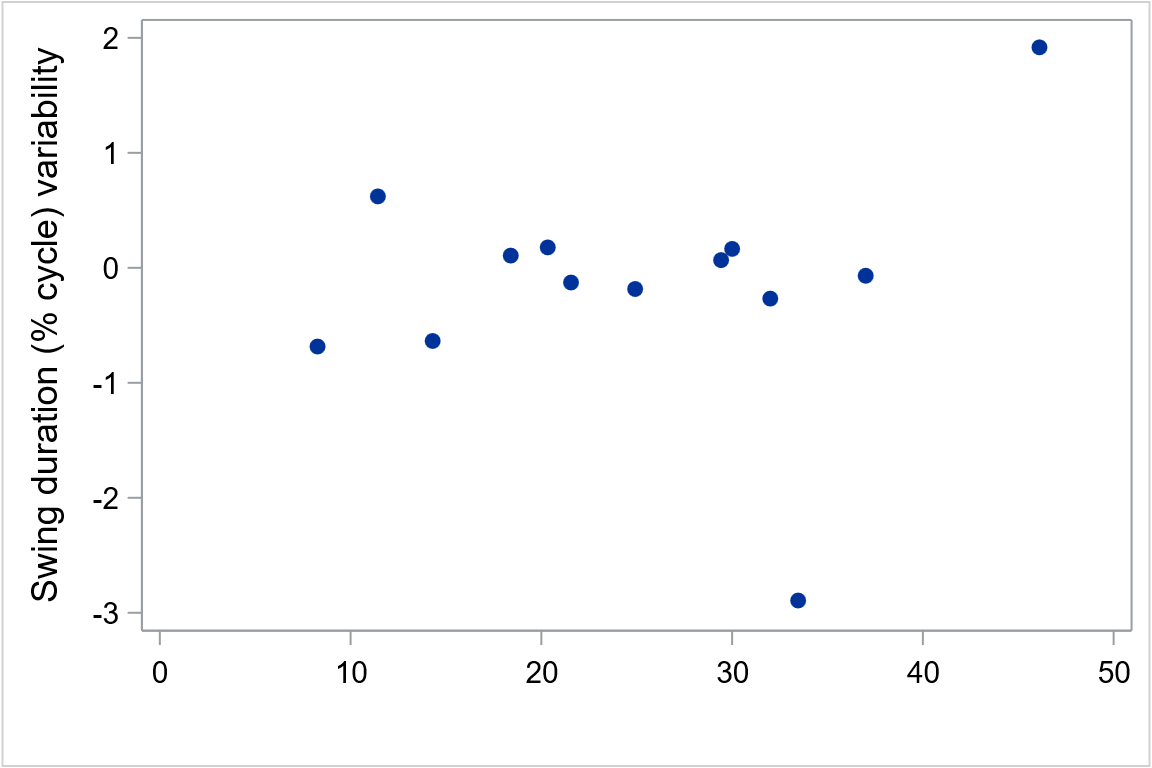

Supplement: Multimedia Appendix 3 [file cancer_v12i1e80909_app3.docx]
